# The effect of perioperative AKI on surgical outcomes

**DOI:** 10.1007/s44254-023-00032-4

**Published:** 2023-09-18

**Authors:** Xisheng Shan, James E. Littlejohn, Ke Peng, Fuhai Ji, Hong Liu

**Affiliations:** 1 Department of Anesthesiology, The First Affiliated Hospital of Soochow University, Suzhou 215006, China; 2 Department of Anesthesiology and Pain Medicine, University of California Davis Health, Sacramento 95817, CA, USA

Postoperative acute kidney injury (AKI) is a common complication that is associated with development of chronic kidney failure, prolonged hospitalization and increased mortality. The occurrence rate ranges from 10 to 47% in high-risk surgeries and patients [1]. The etiology and mechanisms are complex and multifactorial. It has been suggested that even small rises in creatinine (≥ 0.3 mg/dl) are independently related to an approximately fourfold increase in hospital mortality. Hence, it is important to identify the risk factors and the pathophysiology of AKI to develop the strategies to prevent and treat AKI.

In this issue of the journal, Mok and colleagues summarize the pathophysiology, treatment, and surgical outcomes of perioperative AKI, and discuss updated measures of AKI prevention and treatment. This review describes the three main definitions of AKI: the Risk, Injury, Failure, Loss and End-stage kidney disease (RIFLE) criteria, the Acute Kidney Injury Network (AKIN) criteria and the Kidney Disease: Improving Global Outcomes (KDIGO) criteria. Even though the 2012 KDIGO task force offers a cohesive interpretation of RIFLE and AKIN criteria, there is still no consensus on which criteria is more accurate in evaluating AKI. Mok and colleagues also break down common etiologies of AKI into prerenal, renal and postrenal causes (Fig. 1). If the etiology of AKI can be reversed rapidly, renal function can generally be recovered.

The kidney is regarded as a highly vascularized organ, characterized by a remarkable diversity of endothelial cell populations. The function of endothelial cells is increasingly well understood. Molitoris and colleagues describe the renal vascular endothelium as a dynamic organ, which promotes an antithrombotic and anti-inflammatory environment and maintains tissue perfusion and vascular tone under physiological conditions. Impairment of these vital endothelial cell functions contributes to reduced renal perfusion, continued renal hypoxia, and the subsequent diminution in glomerular filtration rate that is the feature of AKI. Mok and colleagues also describe the causes of renal vascular endothelial cell injury, including hypoxia, ischemia or nephrotoxicity. Those pathophysiological processes can be triggered and occur in specific AKI cases simultaneously. This review describes that up to 60% of in-hospital AKI cases are caused by drug-induced nephrotoxicity which includes non-steroidal anti-inflammatory drugs (NSAIDs), aminoglycoside antibiotics and intravenous imaging contrast agents, which should be taken into account in the perioperative management of patients.

Mok and colleagues further discussed perioperative risk factors for AKI. During the intraoperative period, patients who underwent emergency surgery, large volume of blood loss and blood transfusion, re-do surgery, such as liver transplant, more complex procedures, longer surgery duration, and longer cardiopulmonary bypass (CPB) time are predisposed to develop AKI. Therefore, more studies have focused on major surgical procedures such as cardiac surgery and CPB. In fact, special types of surgeries involving major vascular and renal vascular operations, such as liver transplantation and abdominal aortic aneurysm surgery, can also greatly increase the occurrence of AKI [2]. A Meta analysis also indicated that the incidence of post-liver transplant AKI remained stable over the ten years from 2007 to 2018 [3]. Mok and colleagues suggested that patients with perioperative AKI manifest worse short-term and long-term prognoses, which has already been confirmed by published studies. A study in patients with type A acute aortic dissection indicated that AKI was associated with a 249% increase in 30-day mortality [4]. Another study of 8320 cases suggested that patients with postoperative AKI requiring dialysis (AKI-D) at a median follow-up of 294.5 days had a significant higher all-cause long-term mortality compared to controls [5]. The author stated that continuous renal replacement therapy (CRRT) in critically ill patients with unstable hemodynamics may in fact improve the long-term prognosis. Another study showed that patients who received CRRT acutely after surgery had a very low risk of requiring long-term renal replacement therapy (RRT) and had a lower mortality rate compared to patients that received intermittent RRT [6]. Early intervention with CRRT may be beneficial for the recovery of the renal function, which is an independent risk factor for long-term survival. However, it is unclear whether starting RRT earlier would help prevent AKI, even though most clinicians choose to delay RRT as much as possible.

Early renal impairment detection and preoperative risk stratification are vital for early intervention in order to preserve renal function. The authors describe approaches to early AKI detection by using new biomarkers. The serum and urine neutrophil gelatinase-associated lipocalin (NGAL) has been particularly effective at predicting AKI. Serum NGAL is highly sensitive for the early renal injury, but it is not specific for AKI. Other biomarkers so far including cystatin C (Cys-C), N-acetyl-glucosaminidase (NAG), kidney injury molecule 1 (KIM-1), interleukin-6 (IL-6), interleukin-8 (IL-8), interleukin 18 (IL-18), liver-type fatty acid-binding protein (L-FABP), calprotectin, urine angiotensinogen (AGT), urine microRNAs, insulin-like growth factor-binding protein 7 (IGFBP7), and tissue inhibitor of metalloproteinases-2 (TIMP-2) are being explored and they may also play a role in early detection of AKI in specific populations [7]. In view of the complexity of AKI, perhaps the utilization of a panel of several biomarkers across different stages of the syndrome could offer a better understanding of its pathophysiological state. In addition, elevated concentrations of an AKI biomarker without meeting KDIGO classification is defined as subclinical AKI. It is crucial to develop methods to predict patients at high risk for AKI and identify subclinical AKI. Unexpectedly, some early AKI detection methods in the past years such as hospital electronic medical record alert system do not lead to better clinical outcomes. Nevertheless, a recent study by Hodgson et al. combining electronic medical record alert system and care bundle measures demonstrated a decrease in hospital-acquired AKI and a decrease in AKI-associated mortality. Thus, the lack of improvement may attribute to early detection alone without management measures. Furthermore, utilizing perioperative characteristics (such as patient information, intraoperative vital signs, perioperative laboratory tests, etc.) combined with artificial intelligence (AI), namely, machine learning techniques for postoperative AKI prediction is currently the main trend in this type of research [8]. In most studies, the machine learning algorithm performed impressively, with higher sensitivity than specificity in order to detect as many cases of AKI as possible at an early stage. Although there are some medical warning systems for postoperative AKI, the lack of clinical drug treatment methods or strategies still leads to a high incidence of AKI. Other available intervention methods in clinical practice include perioperative adjustment of vasoactive drugs, dietary optimization, pre-adjustment of electrolytes, and so on [9]. Furthermore, the KDIGO guidelines for AKI prevention and treatment is recommended, including administering isotonic crystalloid as the first-line treatment, avoiding or replacing nephrotoxic agents, maintaining normoglycemia, and hemodynamic management. Novel drugs targeting AKI may be further explored in basic research. Recent research has pointed out that hypoxia-inducible factors (HIFs) and extracellular adenosine signaling could facilitate adaptive responses to low oxygen and lead to anti-inflammatory function during acute disease states [10]. New therapeutic pathways targeting these two signaling pathways can be developed to treat or prevent surgical AKI as future directions of the field. Moreover, dexmedetomidine, an α_2_ adrenoreceptor agonist, has demonstrated renal protective properties in both animal experiments and clinical trials. It is also suggested that dexmedetomidine may be a promising agent for the renal protection and further research of pharmacological treatments of AKI in specific population should be conducted in large-scale randomized controlled trials.

In summary, postoperative AKI is a common and severe complication that is associated with adverse outcomes. It is important to understand the pathophysiology of perioperative AKI, the latest prevention and treatment concepts, and the impact on surgical outcome. Mok and colleagues provide an in-depth review on all aspects of perioperative AKI. However, the prevalence AKI is still high despite of these efforts. It has been suggested that further research of pharmacological treatments of AKI should be conducted in large-scale randomized controlled trials representative of the diverse population affected by AKI.

## Figures and Tables

**Fig. 1:**
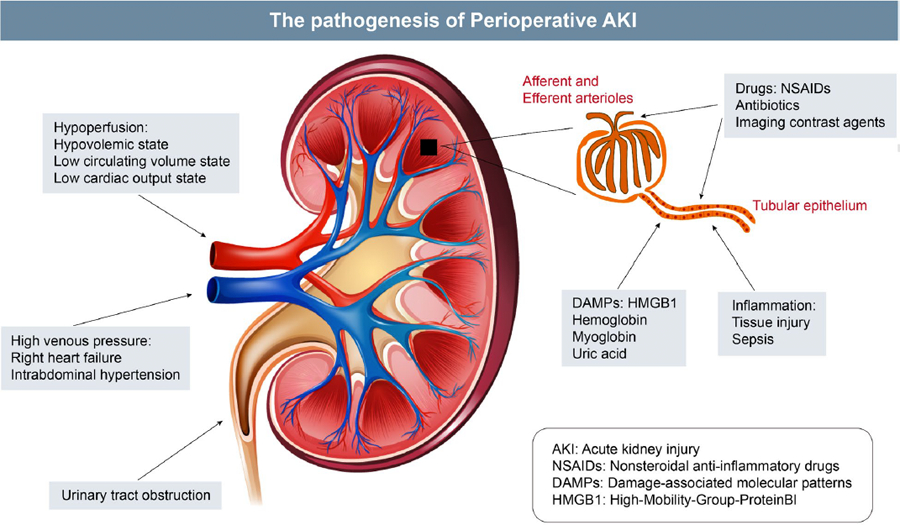
Pathogenesis of perioperative acute kidney injury (AKI). NSAIDs, nonsteroidal anti-inflammatory drugs, DAMPs, damage-associated molecular patterns, HMGB1, high-mobility-group-protein B1

## Data Availability

Not applicable.

## Abbreviations

AGT: Urine angiotensinogen
AI: Artificial intelligence
AKI: Acute kidney injury
AKI-D: Acute kidney injury requiring dialysis
AKIN: Acute kidney injury network
CPB: Cardiopulmonary bypass
CRRT: Continuous renal replacement therapy
Cys-C: Cystatin C
DAMPs: Damage-associated molecular patterns
HIFs: Hypoxia-inducible factors
HMGB1: High-mobility-group-protein B1
IGFBP7: Insulin-like growth factor-binding protein 7
IL-6: Interleukin-6
IL-8: Interleukin-8
IL-18: Interleukin-18
KDIGO: Kidney disease: improving global outcomes
KIM-1: Kidney injury molecule 1
L-FABP: Liver-type fatty acid-binding protein
NAG: N-acetyl-glucosaminidase
NGAL: Neutrophil gelatinase-associated lipocalin
NSAIDs: Non-steroidal anti-inflammatory drugs
RIFLE: Risk, injury, failure, loss and end-stage kidney disease
RRT: Renal replacement therapy
TIMP-2: Tissue inhibitor of metalloproteinases-2
